# The use of accelerometer bracelets to evaluate arm motor function over a stroke rehabilitation period – an explorative observational study

**DOI:** 10.1186/s12984-024-01381-2

**Published:** 2024-05-20

**Authors:** Eric Lyckegård Finn, Håkan Carlsson, Petter Ericson, Kalle Åström, Christina Brogårdh, Johan Wasselius

**Affiliations:** 1 Uman Sense AB, Lund, Sweden; 2 Department of Neurology, Rehabilitation Medicine, Memory Disorders and Geriatrics, Skåne University Hospital, Lund, Sweden; 3 Department of Health Sciences, Lund University, Lund, Sweden; 4 Centre for Mathematical Sciences, Lund University, Lund, Sweden; 5 Department of Medical Imaging and Physiology, Skåne University Hospital, Lund, 221 85 Sweden; 6 Department of Clinical Sciences Lund, Lund University, Lund, Sweden

**Keywords:** Stroke rehabilitation, Accelerometry, The modified motor assessment scale, The motor activity log, Arm motor activity, Wrist-worn accelerometers, Sensors

## Abstract

**Background:**

Assessments of arm motor function are usually based on clinical examinations or self-reported rating scales. Wrist-worn accelerometers can be a good complement to measure movement patterns after stroke. Currently there is limited knowledge of how accelerometry correlate to clinically used scales. The purpose of this study was therefore to evaluate the relationship between intermittent measurements of wrist-worn accelerometers and the patient’s progression of arm motor function assessed by routine clinical outcome measures during a rehabilitation period.

**Methods:**

Patients enrolled in in-hospital rehabilitation following a stroke were invited. Included patients were asked to wear wrist accelerometers for 24 h at the start (T1) and end (T2) of their rehabilitation period. On both occasions arm motor function was assessed by the modified Motor Assessment Scale (M_MAS) and the Motor Activity Log (MAL). The recorded accelerometry was compared to M_MAS and MAL.

**Results:**

20 patients were included, of which 18 completed all measurements and were therefore included in the final analysis. The resulting Spearman’s rank correlation coefficient showed a strong positive correlation between measured wrist acceleration in the affected arm and M-MAS and MAL values at T1, 0.94 (*p* < 0.05) for M_MAS and 0.74 (*p* < 0.05) for the MAL values, and a slightly weaker positive correlation at T2, 0.57 (*p* < 0.05) for M_MAS and 0.46 − 0.45 (*p* = 0.06) for the MAL values. However, no correlation was seen for the difference between the two sessions.

**Conclusions:**

The results confirm that the wrist acceleration can differentiate between the affected and non-affected arm, and that there is a positive correlation between accelerometry and clinical measures. Many of the patients did not change their M-MAS or MAL scores during the rehabilitation period, which may explain why no correlation was seen for the difference between measurements during the rehabilitation period. Further studies should include continuous accelerometry throughout the rehabilitation period to reduce the impact of day-to-day variability.

## Introduction

Assessment of motor function and motion pattern is a central part of evaluation of neurological conditions. Currently, such evaluation is largely based on self-reported rating scales and clinical examination by healthcare professionals. However, clinical examination is limited to assessing impairments or activity limitations but not the motion pattern in an objective way. Another limiting factor is that currently it is not possible to evaluate the patients in an in-home environment apart from self-reporting rating scales.

With the introduction of comfortable and affordable wearable sensors, detailed kinetic data can be a useful complement to clinical outcome measures, and continuously recorded with a low level of intrusiveness for the patient in an in-home environment. Such sensors include accelerometers [1–4], EMG sensors [5, 6] and magnets [7]. Sensors can be worn on the body [1, 8, 9], integrated in bracelets [2, 3, 7], or in gloves [10]. Sensors can be used for many purposes, such as activity monitoring of elderly [11], oncology patients [12], or to monitor vital signs in surgical patients [13].

One field where measurement of motor function with sensors is of great importance is within stroke rehabilitation, where long-term improvements may be difficult to quantify and separate from compensatory movement patterns and a learned non-use behavior [14]. Wearable sensors have been used within rehabilitation to monitor gait [8, 15], fall risk [1] and arm motor function [1, 6, 7] and are shown to be appreciated by rehabilitation professionals as well as by patients [16]. Sensors have also been combined with in-app training programs for home rehabilitation [2, 17] of chronic stroke patients to improve participation. Such in-app training programs can help measure and improve adherence to the training by providing instructions and guidance, but also providing an accessible communication platform with the rehabilitation team. Potentially, it could measure the training dose as well as the use of the affected arm in daily activities during the rehabilitation.

Sensors have also been used in stroke care, for diagnostic purposes by identifying unliteral arm motor deficit [3, 18–20], and for monitoring and evaluation of rehabilitation following stroke [21–24]. However, a study by Rand et al. 2012 on 60 stroke patients receiving rehabilitation, showed that upper extremity activity measured by activity monitors was not correlated to increased functional status of the affected arm [21]. Similar result has been seen in several other studies where functional recovery has not been correlated to increased arm movement as measured by accelerometry [23, 24]. Contradictory to this, Gohlke et al. showed that functional recovery was associated with increased arm movement when measured by bilateral wrist accelerometry at multiple times during the rehabilitation of 14 stroke patients [22]. Since the results of the various studies differ, more studies are needed within this area.

The purpose of this study was to evaluate the relationship between intermittent measurements of wrist-worn accelerometers and the patient’s progression of arm motor function as assessed by the clinical outcome measures Motor Assessment Scale (MAS) [25, 26] and Motor Activity Log (MAL) [27, 28] during an active rehabilitation period.

Our hypothesis was that an improvement in MAL and MAS would correlate with an increase in upper limb activity, as measured by the accelerometers, and normalization in the balance between right and left arm activity.

## Materials and methods

### Study population

Patients referred to the inpatient rehabilitation unit at Skåne University Hospital in the subacute phase after stroke were recruited to the study between January 2018 and February 2020.

Inclusion criteria were:

- Recent stroke with unilateral arm motor deficit, AND;
- Ongoing rehabilitation, AND;
- No previous condition affecting the arm motor function of the unaffected arm.

Exclusion criteria were:

- Age younger than 18, OR;
- Inability to give informed consent, OR;
- Unwillingness to participate.

Before inclusion in the study, all participants received verbal and written information about the purpose of the study and gave written consent to participate. The local Ethical committee approved the study (#2015 − 387), and all study activities were conducted in accordance with the declaration of Helsinki.

### Data collection

A physiotherapist (HC) working at the rehabilitation unit performed the assessments at the beginning of the rehabilitation period (test occasion 1; T1) and at the end of the rehabilitation period (test occasion 2; T2). All participants underwent a team-based inpatient rehabilitation with person-centered training based on their needs and goals. They received 2 to 3 h of training, 5 days a week by a physiotherapist and an occupational therapist. The training consisted of task-specific training in functional tasks, active and passive arm movements, strength training and stretching.

### Accelerometer data collection

Motion data were recorded for 24 h at both T1 and T2 using triaxial accelerometer bracelets on both wrists (E4, Empatica Inc, Cambridge, MA, USA). Accelerometer data were collected and downloaded from the bracelets using the manufacturer’s software (E4 manager, Empatica Inc, Cambridge, MA, USA). Downloaded data included date and time, triaxial accelerometer data sampled at 32 Hz and additional sensor data not used here (4 Hz Electrodermal Activity sensor (EDA), 4 Hz Infrared Thermopile sensor and 64 Hz Photopletysmogram sensor (PPG)). Data was further processed using MATLAB (Mathworks, Cambridge, MA, USA).

### Clinical assessment of arm motor function

Two outcome measures were used to assess arm motor function at T1 and T2: (i) the modified Motor Assessment Scale (MAS) [25, 26, 29]), and (ii) the Motor Activity Log (MAL) [30].

#### The modified motor assessment scale

In the modified Motor Assessment Scale [26, 29], the three domains assessing gross arm motor function, hand motor function, and advanced hand motor function (i.e., dexterity) were used. These domains include 15 items, where the participant’s ability to complete a task on time and the quality of movement was assessed on a 0–5-point scale. Both arms were tested separately, and the total sum score for each arm ranged from 0 to 15 points. Higher scores indicate greater ability and arm motor function. Only the score for the stroke affected arm was later used in the comparison. The scale has been tested for validity and reliability in Swedish [26, 29].

#### Motor activity Log

The Motor Activity Log is a rating scale where participants are asked to assess how often (amount of use, AoU) and how well (quality of movement, QoM) they can use the affected hand in 30 daily activities [27, 28]. The AoU scale ranges from 0 (never use the more affected arm for the activity) to 5 (always use the more affected arm for the activity), and the QoM scale ranges from 0 (inability to use the more affected arm for the activity) to 5 (ability to use the more affected arm for the activity just as well as before the stroke). The total score of each subscale ranges from 0 to 150 points, which is divided by 30, resulting in a mean score. Higher scores indicate greater ability and arm motor function. The MAL has been shown to be valid and reliable after stroke [31].

### Accelerometer data processing

#### Data pre-processing

The collected tri-axial accelerometer data in the 8-bit range − 128 to 127, where a value of 64 was equal to Earths gravitational constant 9.82 m/s^2, was pre-processed in the same way as in [3]. The collected arm movement data was first filtered through a 5th order Butterworth high-pass filter with a cut off frequency of 3 Hz. Only the magnitude of the accelerometer signal was of interest and therefore Euclidian norm was calculated for the tri-axial accelerometer signal resulting in a single positive value representing the magnitude of the acceleration for each sample point. The amount of data was then reduced using a moving average filter over 96 samples followed by a subsampling of 48 resulting in sampling frequency reduction to 0.67 Hz compared with the original 32 Hz sampling frequency. This helped smooth the data and made the amount data more manageable as an entire 24 period had been collected at each recording session.

The signals from the two arms were collected independently using two separate wristbands which resulted in a small sync error between the two arms because of the wristbands being powered on at slightly different times. The sync error was corrected by identifying the optimal overlap between the two signals where the sum of the differences of the measurements from the two arms were minimized. In addition, long periods of zero data at the end of a collection period caused by the bracelets not being powered off at the time when they were taken off and placed on a still surface were manually removed.

#### Extracting the most active period

To remove the impact of the amount of sleep during the day of measurement and to focus on the time when the patient was most active with their arms, only the top 10% most active sample-pairs were selected from each recording. The sum of the two arms at a given point determined the activity of a sample pair, where a higher sum was equivalent to more activity. The top 10% most active sample pairs were extracted for each recording session, and this was equivalent to extracting roughly the most active 2.5 h period of the day for a 24 h sample recording. The mean value of each of the two arms from the extracted period was then calculated and used as the recorded arm acceleration value for each arm.

## Results

### Study population

A total of 20 patients (14 men and 6 women) were included in the study. Two of them failed to complete both recording sessions. One patient left the rehabilitation program before completing the final measurement and assessments at T2, and the other patient had a technical malfunction, where one bracelet accidentally was not started at the recording session and unfortunately the patient had left the rehabilitation clinic when the mistake was discovered and could not be redone. Thus, data from 18 patients were used in the final analysis.

The characteristics of the 18 participants that completed both recordings and assessment sessions are shown in Table 1. On average, the length of the rehabilitation period was 29.6 days (SD ± 9.6).

**Table 1:** Patient characteristics and stroke characteristics for the final study population

**Patient characteristics**		
Age, years; median (range)		58.5 (27–72)
Gender; male/female		13/5
**Stroke characteristics**		
Stroke type (ischemic/hemorrhagic)		8/10
Time since stroke (months); mean (± SD)		1.6 (± 1)
Pre-stroke mRS, median (range)		0 (0–0)
NIHSS, median		10 (4–40)
Side of paresis; right/left		8/10
Arm weakness (NIHSS), median (range)		3 (1–4)
mRS @ 3months	0–2	0
3		5
4		13
5		0

SD = Standard Deviation, mRS = modified Rankin Scale, NIHSS = National Institute of Health Stroke Scale

### Arm motor function at the two test occasions

Data of arm motor function for the more affected arm before (T1) and at the end of the rehabilitation period (T2) are presented in Table 2.

**Table 2:** Data for the 18 participants at Test occasion 1 (before the rehabilitation period) and 2 (at the end of the rehabilitation period)

Variable	Test occasion 1	Test occasion 2
**MAS** (0-15p) Median (Q1-Q3)	3.0 (0.0-7.7)	7.5 (0.00-12.25)
**MAL AOU** (0-5p) Median (Q1-Q3)	0.05 (0.00-1.52)	0.18 (0.00-2.18)
**MAL QOM** (0-5p) Median (Q1-Q3)	0.03 (0.00-1.46)	0.17 (0.00-2.11)
**Acceleration (m/s** ^**2**^ **)** Median (Q1-Q3)	1.43 (1.01–1.74)	1.72 (1.23–2.15)

MAS = Modified Motor Assessment Scale, MAL AOU = Motor Activity Log Amount of Use, MAL QOM = Motor Activity Log Quality of Movement

**Fig. 1:**
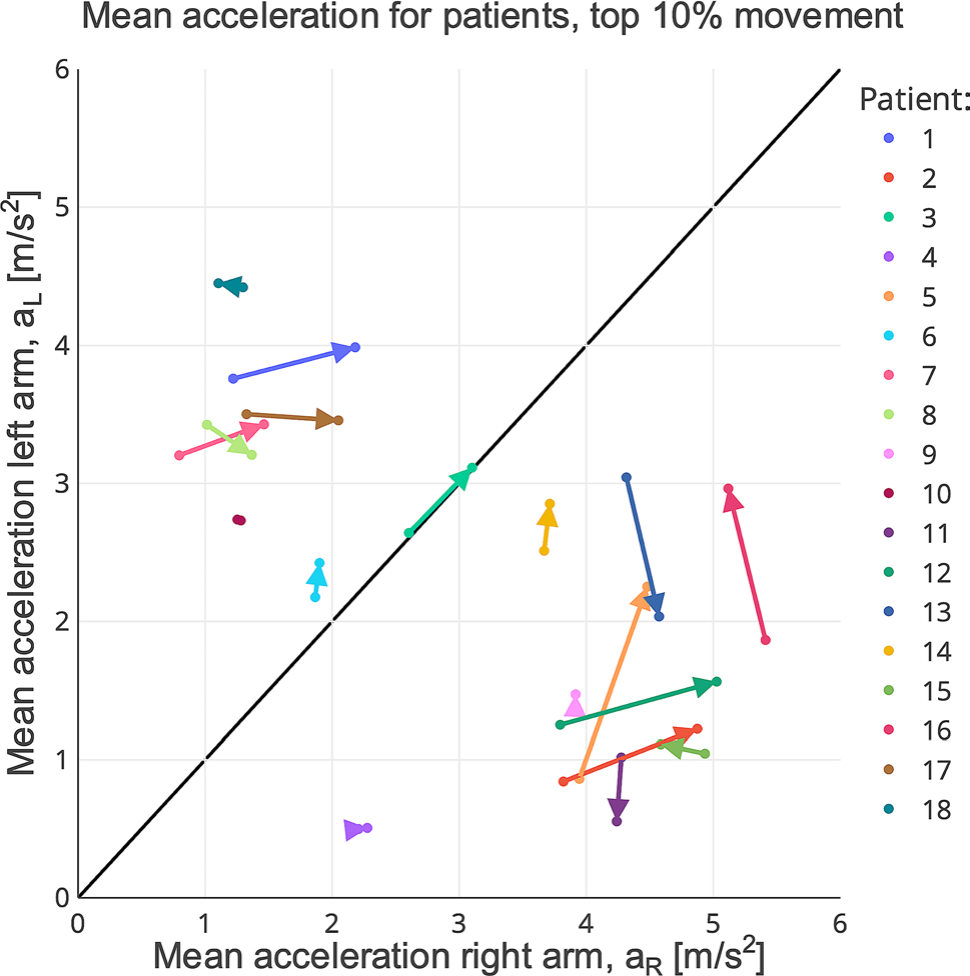
The measured mean acceleration for the top 10% largest arm movement pairs for all 18 patients who completed both measurement sessions (T1 and T2). Each subject is plotted with a unique color and the two measuring sessions T1 and T2 have been linked with an arrow pointing from T1 to T2. The closer a subject is to the diagonal line the more symmetrical is the movement recorded by the accelerometer on the right and left wrist. A value further away from the origin point illustrates a larger overall movement compared with a value closer to the origin point

The measured mean acceleration for the 10% most active arm movement pairs for each patient and test occasion can be seen Fig. 1. Each color corresponds to one patient, and data from the two test occasions (T1 and T2) have been linked together, where the arrow points from T1 to T2. As can be seen, most patients are located away from the symmetry line, either above the line if they are weaker in their right arm or below the line if they are weaker in their left arm. As the diagonal line represents perfect symmetry between the two arms, patients are expected to be closer to the symmetry line at T2 compared to T1, which means that the arrow should point towards the symmetry line. This can be observed for some of the patients, but not for all.

**Fig. 2:**
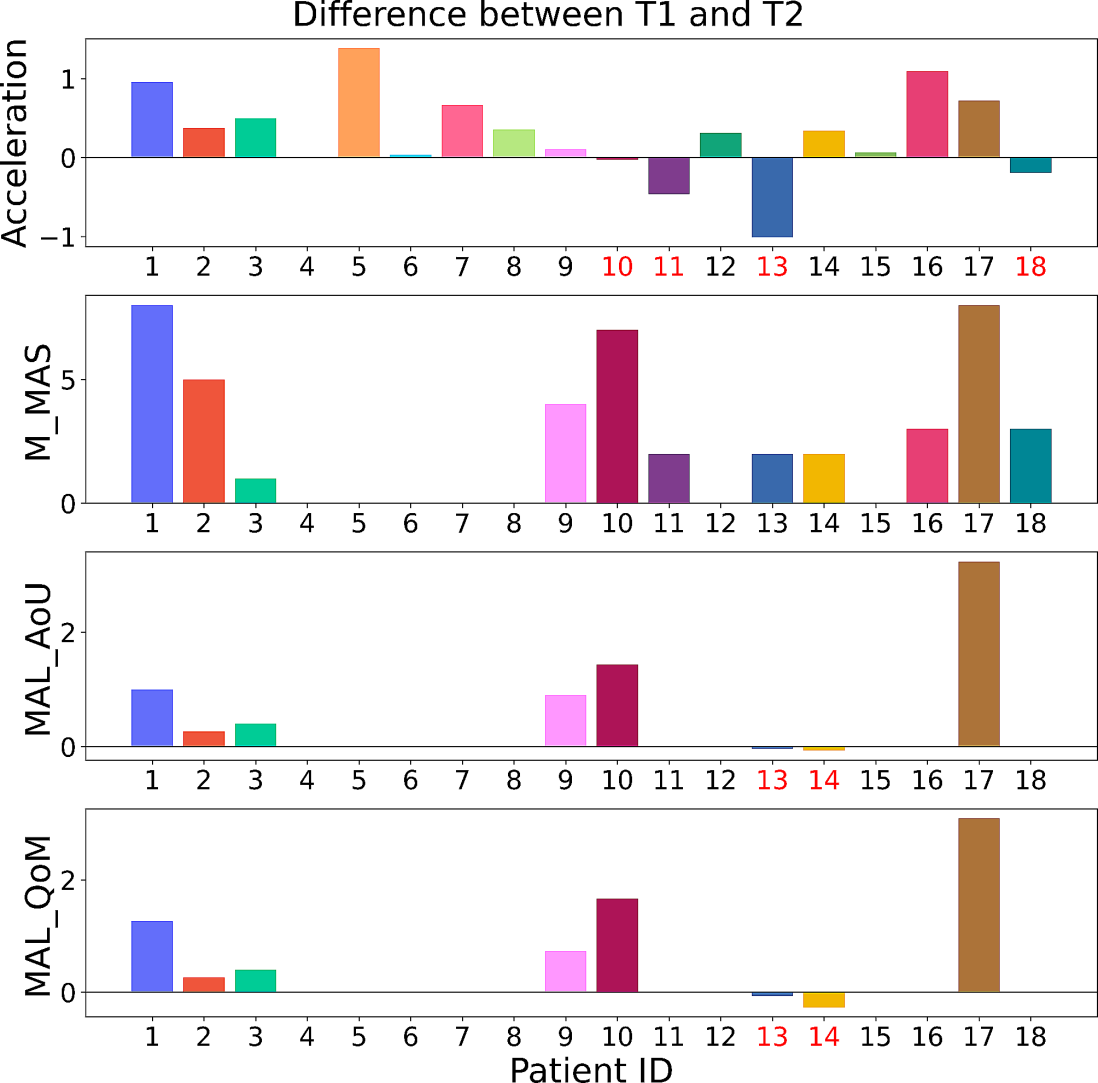
Difference in measured acceleration of the affected arm, MAS, MAL_AoU and MAL_QoM between test session T1 and T2 for each patient that successfully completed two test sessions. Each bar is colored in a color unique to each patient with corresponding patient numbers located on the x-axis. Bars with red label text on the x-axis correspond to a negative progression between session T1 and T2

Figure 2 shows the difference in mean acceleration, Motor Assessment Scale (MAS), Motor Activity Log Amount of Use (MAL_AoU) and Motor Activity Log Quality of Movement (MAL_QoM) for each patient. A positive value in Fig. 2 is equivalent to an improvement in the specific metric, a value of 0 is equal to no difference between T1 and T2 and a negative value means that the patient obtained a lower score at T2 compared with the initial T1.

**Fig. 3:**
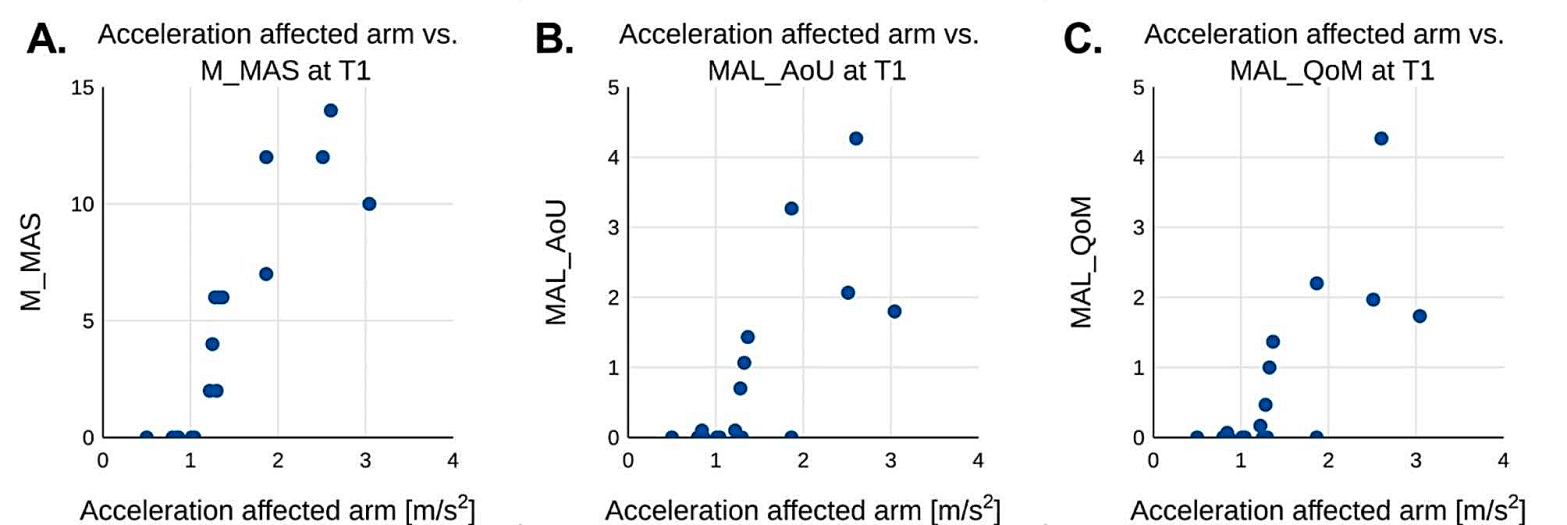
The figure shows the scores from M_MAS (**A**), MAL_AoU (**B**) and MAL_QoM (**C**) plotted against the average recorded acceleration for the affected arm at the first test session T1

**Fig. 4:**
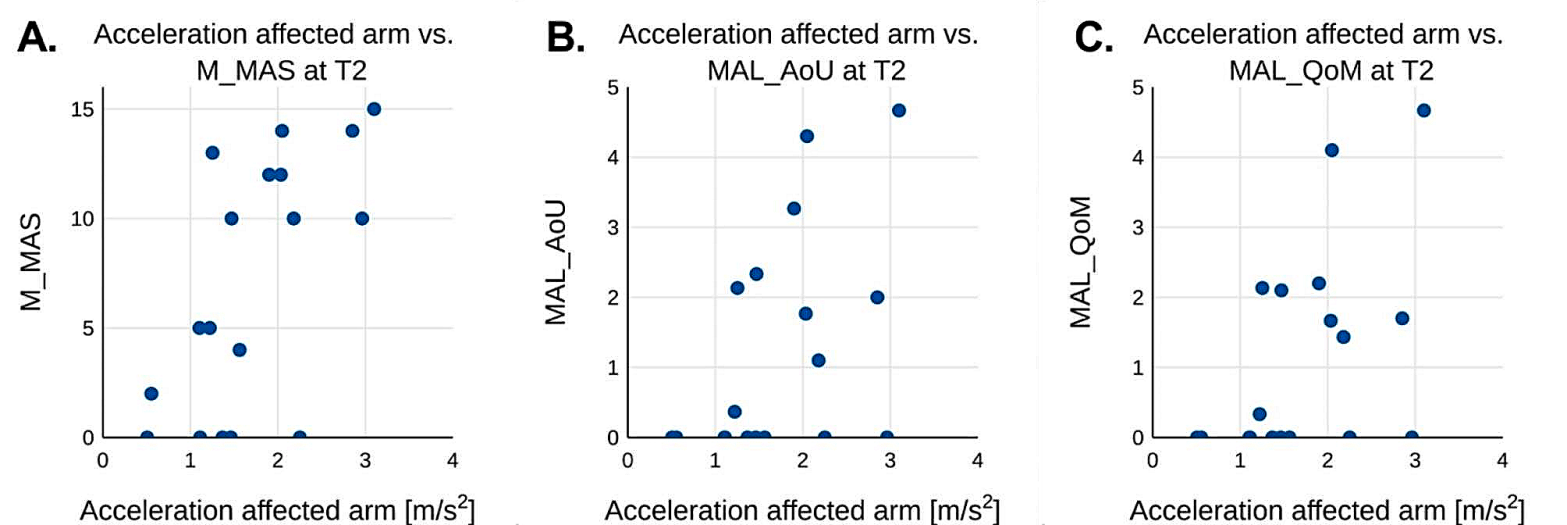
Shows the scores from M_MAS (**A**), MAL_AoU (**B**) and MAL_QoM (**C**) plotted against the average recorded acceleration for the affected arm at the second test session T2

**Fig. 5:**
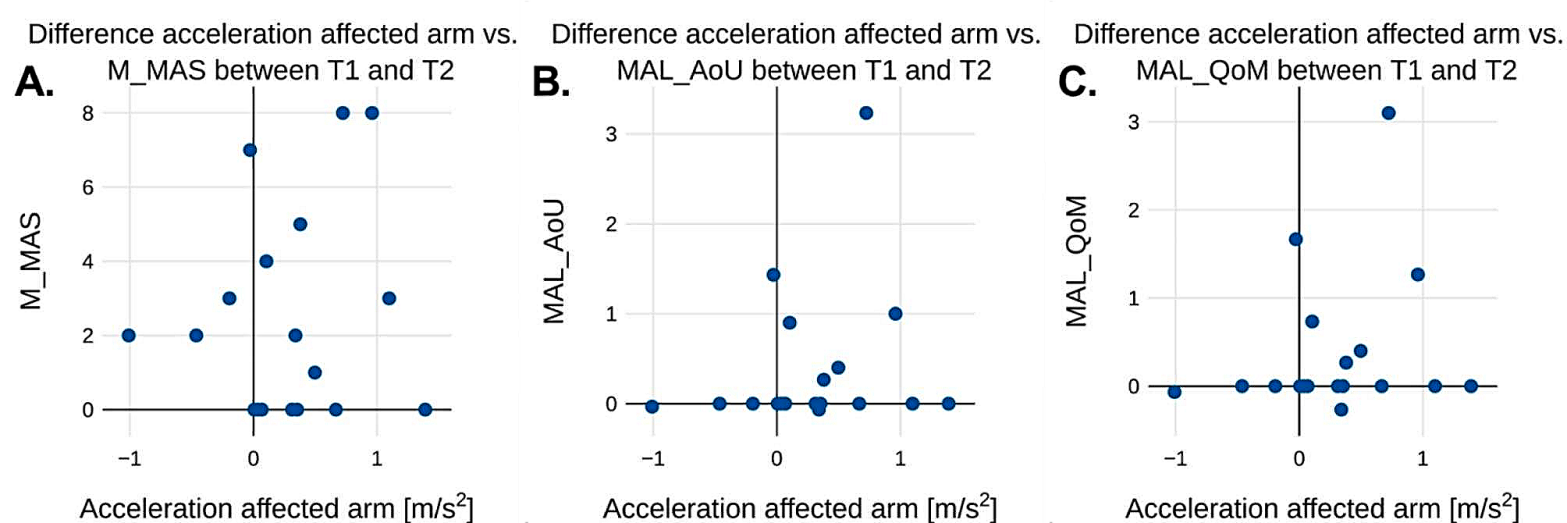
The calculated difference in M_MAS (**A**), MAL_AoU (**B**) and MAL_QoM (**C**) against the difference in average recorded acceleration between session T1 and T2 for the affected arm

Figure 3a-c shows the mean acceleration values for each patient during T1 plotted against either the M_MAS value (3a), MAL_AoU (3b) or MAL_QoM obtained at T1. Fig. 4a-c illustrates the corresponding values for T2. Fig. 5a-c shows the difference in mean acceleration and one of the three metrics M_MAS (5a), MAL_AoU (5b), or MAL_QoM between T1 and T2 for each patient.

Table 3 contains the calculated correlations between the pairwise comparison of the mean acceleration and any of the other three measured values. The correlations have been calculated using the Spearman’s ranked correlation test as it does not assume the values to be normally distributed.

**Table 3:** Correlation analysis between Accelerometry of the affected arm and MAS, MAL_AoU and MAL_QoM at Test occasion 1 (T1), Test occasion 2 (T2) and the differences between T1 and T2 (∆ T1-T2)

Correlation analysis between:	Spearman’s rank correlation coefficient (*p*-value)		
	T1	T2	∆ T1-T2
Acceleration affected arm vs. MAS	0.94 (< 0.05)	0.57 (< 0.05)	0.08 (0.75)
Acceleration affected arm vs. MAL_AoU	0.74 (< 0.05)	0.45 (0.06)	0.29 (0.24)
Acceleration affected arm vs. MAL_QoM	0.73 (< 0.05)	0.46 (0.06)	0.29 (0.24)

MAS = Modified Motor Assessment Scale, MAL_AOU = Motor Activity Log Amount of Use, MAL_QOM = Motor Activity Log Quality of Movement

## Discussion

In this explorative observational study, we compared 24 h accelerometry with measures of arm motor function used in clinical rehabilitation practice in patients in the beginning and end of their rehabilitation program following a stroke. The patients tolerated the devices well and there was only one case of technical failure at one registration session.

The measured wrist acceleration on the affected arm correlated with measurements of arm motor function (MAS, MAL_AoU and MAL_QoM) as seen in Table 3 as well as seen in Figs. 3 and 4 at both T1 and T2. At T1 there was a very strong correlation between M_MAS and wrist acceleration with a calculated Spearman’s ranked correlation coefficient of 0.94. The correlation for MAL_AoU and MAL_QoM were slightly lower at 0.74 and 0.73 respectively.

A positive correlation was also observed at T2 between wrist acceleration and MAS, although lower than at T1 with a Spearman’s correlation coefficient of 0.57. For MAL_AoU and MAL_QoM correlations were also lower than T1 with coefficients of 0.45 and 0.46, and these values were just outside the limit for being statistically significant with a p-value of 0.06.

A possible reason for the fact that the correlation was stronger between accelerometry and M_MAS at T1 compared with MAL_AoU and MAL_QoM may be that patients had difficulties to self-estimate their arm function early on in their rehabilitation, as they have not yet had time to properly use their affected arm in daily activities, which is in line with other studies [Hussain 2020]. Although the correlations increased over time between Abilhand and other objective measurements in the study by [32], a decrease in strength of the correlations was found for 3 out of 4 calculations between day 10 and week 4, which is similar to the results observed here during a similar time frame.

The measured correlation between acceleration and MAL_AoU and MAL_QoM are also similar in range to that obtained in other studies [19, 20]. In the study by van der Pas, 45 stroke patients were measured for three consecutive days [19], and in the study by Narai 19 stroke patients wore bilateral triaxial accelerometer bracelets over a 24-hour period [20], similar to the first measurement in our study. In both studies they observed a correlation between MAL and wrist acceleration in between those measured at T1 and T2 which is likely explained by the fact that they had a broader inclusion with regard to time between stroke occurrence and inclusion into the study.

Since a positive correlation was observed at both T1 and T2 between wrist accelerometry and the physical measurements of arm motor function, one would expect to see a similar positive correlation when comparing the delta values between T1 and T2. If scores of M_MAS, MAL_AoU or MAL_QoM are improved between T1 and T2, it would be expected that the wrist accelerometry would improve as well due to the positive correlation. However, as seen in Fig. 5; Table 3 there are no observed statistically significant correlation between the delta values. This is an interesting result which may have several possible explanations.

One contributing factor to the unobserved correlation among the delta values could be that many of the patients obtained the same results in both MAL and M_MAS for T1 and T2. In fact, 10 out of 18 patients achieved the same MAL_AoU and MAL_QoM results and 11 out of 18 achieved the same M_MAS results, see Table 2. At the same time, most patients recorded some difference in accelerometry value as it is unlikely to measure the exact same accelerometry value twice since it is a continuous value instead of discrete. The low improvement in M_MAS and MAL for many of the patients could be due to that they were quite severely affected in their weak arm with low median values in both outcome measures (see Table 2) compared to other similar studies [33]. The 18 patients in our study had a lower score in M_MAS and MAL compared to the 30 patients in the study by Hammer and Lindmark [33], and showed less improvement in MAL and similar improvement in M_MAS.

Another contributing factor to the low correlations for the delta values between the two test sessions could be that there is a potential variability in measured acceleration for a single individual between different days not caused by arm motor deficit or rehabilitation. It is most likely that arm acceleration will to some degree depend on how active the patient was during the day, and lower level of acceleration at T2 compared with T1 for four of the patients, as seen in Figs. 2 and 5, could be a reflection of this. To obtain more robust results it would be of interest to record wrist acceleration continuously throughout rehabilitation. By doing so it would be possible to analyze the trend in level of recorded movement thus removing the impact of any potential variability between days.

From Fig. 1 it can be seen that the measured wrist acceleration is able to separate the patients as either being weak in the left arm – placing their arrow below the symmetry line, or weaker in their right arm – placing them above the symmetry line. The side of weakness derived from Fig. 1 corresponds perfectly with the weak arm as a result of stroke showing that the accelerometers are able to determine the correct side of weakness. This is in line with previous results showing that accelerometers can be used to separate healthy control individuals from individuals suffering from unilateral weakness [18].

Lastly, another observation that can be seen in Fig. 2 is that several patients improve in accelerometry in the affected arm without any improvement in M_MAS (for example patients 7, 9 and 10). This phenomenon is somewhat counter-intuitive, but previous studies have shown correlation between accelerometry and functional measurements [22] as well as the lack thereof [21], suggesting that accelerometry may not necessary be an alternative, but rather an addition, to functional outcome measures.

Wearable sensors such as accelerometers are now readily available to use in clinical practice for monitoring stroke patients undergoing rehabilitation. Accelerometry may contribute objective information about the objective arm movement over long periods of time. Continuous measurement over the entire rehabilitation period and analyzing trends over time may overcome some of the limitations of short time registration such as in this study. Future work should therefore aim for continuous accelerometry over the entire rehabilitation period and should also aim to include larger populations to overcome individual variability and include the entire range of arm motor disabilities.

The fact that MAL is a self-reported outcome measure of daily arm use and quality of movements, may make it less suitable for comparison with accelerometry since other aspects of functional improvement than physical movements are assessed, such as adjustments, learned ways to compensate for the deficit, or simply accepting the new level of motor function. Other objective measures, such as Fugl-Meyer Assessment (FMA) of Upper Extremity or Action Research Arm Test (ARAT) may be more appropriate in relation to accelerometry. However, combining accelerometry with functional and subjective outcome measures may provide a more comprehensive and complete assessment of the individual rehabilitation process and allow the team to adjust the training to achieve maximal objective as well as subjective improvement.

### Limitations

This study has several limitations. Only patients at one rehabilitation clinic were included and only one type sensor bracelets were used. The second limitation is that the length of the rehabilitation periods was generally shorter than expected, which may have affected the magnitude of improvement. A third limitation is that the study population is small and not based on a preceding power-estimation. A fourth limitation is that the patients in this study generally had severe deficits from their stroke, and the study may not reflect the wide variety of deficits seen post stroke. A fifth limitation is that we chose to use only the 10% of data during each registered 24 h period. This method was chosen to reduce the effect of different daily routines between the two compared time-points and focus only on the samples containing the most movement, but by doing this it is possible that some aspects of accelerometry are lost. On the same note, calculating the Euclidean norm to obtain a single value representing the magnitude of acceleration is a common practice but reduced the data and may affect the result.

## Conclusion

The results confirm that there is a positive correlation between accelerometry and clinical measures and that the wrist acceleration can differentiate between the affected and non-affected arm. Many of the patients did not change their M-MAS or MAL scores during the rehabilitation period, which may explain why no correlation was seen for the difference between measurements during the rehabilitation period. Further studies should include continuous accelerometry throughout the rehabilitation period to reduce the impact of day-to-day variability.

## Data Availability

The datasets used and analyzed during the current study are available from the corresponding author on reasonable request.

## Abbreviations

M_MAS: The Modified Motor Assessment Scale
MAL: The Motor Activity Log
T1: Test occasion 1/beginning of Rehabilitation Period
T2: Test Occasion 2/end of Rehabilitation Period
EMG: Electromyography
Hz: Hertz
AoU: Amount of Use
QoM: Quality of Movement
SD: Standard Deviation
